# The effectiveness of Robot-Assisted Gait Training versus conventional therapy on mobility in severely disabled progressIve MultiplE sclerosis patients (RAGTIME): study protocol for a randomized controlled trial

**DOI:** 10.1186/s13063-017-1838-2

**Published:** 2017-02-27

**Authors:** Sofia Straudi, Fabio Manfredini, Nicola Lamberti, Paolo Zamboni, Francesco Bernardi, Giovanna Marchetti, Paolo Pinton, Massimo Bonora, Paola Secchiero, Veronica Tisato, Stefano Volpato, Nino Basaglia

**Affiliations:** 1 Neuroscience and Rehabilitation Department, Ferrara University Hospital, Via Aldo Moro 8, 44124 Ferrara, Italy; 2 Department of Biomedical and Specialty Surgical Sciences, University of Ferrara, Ferrara, Italy; 3 Unit of Translational Surgery and Vascular Diseases Center, Ferrara University Hospital, Ferrara, Italy; 4 Department of Life Sciences and Biotechnology, University of Ferrara, Ferrara, Italy; 5 Department of Morphology, Surgery and Experimental Medicine, Section of Pathology, Oncology and Experimental Biology, Laboratory for Technologies of Advanced Therapies (LTTA), University of Ferrara, Ferrara, Italy; 6 Department of Morphology, Surgery and Experimental Medicine, Section of Anatomy and Histology, Laboratory for Technologies of Advanced Therapies (LTTA), University of Ferrara, Ferrara, Italy; 7 Center for Clinical Epidemiology, School of Medicine, University of Ferrara, Ferrara, Italy

**Keywords:** Robot-assisted gait training, Progressive multiple sclerosis, Mobility, Motor recovery, Biological markers, Rehabilitation, Plasticity

## Abstract

**Background:**

Gait and mobility impairments affect the quality of life (QoL) of patients with progressive multiple sclerosis (MS). Robot-assisted gait training (RAGT) is an effective rehabilitative treatment but evidence of its superiority compared to other options is lacking. Furthermore, the response to rehabilitation is multidimensional, person-specific and possibly involves functional reorganization processes. The aims of this study are: (1) to test the effectiveness on gait speed, mobility, balance, fatigue and QoL of RAGT compared to conventional therapy (CT) in progressive MS and (2) to explore changes of clinical and circulating biomarkers of neural plasticity.

**Methods:**

This will be a parallel-group, randomized controlled trial design with the assessor blinded to the group allocation of participants. Ninety-eight (49 per arm) progressive MS patients (EDSS scale 6–7) will be randomly assigned to receive twelve 2-h training sessions over a 4-week period (three sessions/week) of either: (1) RAGT intervention on a robotic-driven gait orthosis (Lokomat, Hocoma, Switzerland). The training parameters (torque of the knee and hip drives, treadmill speed, body weight support) are set during the first session and progressively adjusted during training progression or (2) individual conventional physiotherapy focusing on over-ground walking training performed with the habitual walking device. The same assessors will perform outcome measurements at four time points: baseline (before the first intervention session); intermediate (after six training sessions); end of treatment (after the completion of 12 sessions); and follow-up (after 3 months from the end of the training program). The primary outcome is gait speed, assessed by the Timed 25-Foot Walk Test. We will also assess walking endurance, balance, depression, fatigue and QoL as well as instrumental laboratory markers (muscle metabolism, cerebral venous hemodynamics, cortical activation) and circulating laboratory markers (rare circulating cell populations pro and anti-inflammatory cytokines/chemokines, growth factors, neurotrophic factors, coagulation factors, other plasma proteins suggested by transcriptomic analysis and metabolic parameters).

**Discussion:**

The RAGT training is expected to improve mobility compared to the active control intervention in progressive MS. Unique to this study is the analysis of various potential markers of plasticity in relation with clinical outcomes.

**Trial registration:**

ClinicalTrials.gov, identifier: NCT02421731. Registered on 19 January 2015 (retrospectively registered).

**Electronic supplementary material:**

The online version of this article (doi:10.1186/s13063-017-1838-2) contains supplementary material, which is available to authorized users.

## Background

Multiple sclerosis (MS) is a chronic inflammatory disease causing widespread degeneration of the central nervous system. The disease, with different features and progression according to the clinical phenotype [1], gradually results in severe neurological deficits [2] with complex, variable and unpredictable patterns of symptoms [2] including different motor deficits [1]. Locomotor disability and balance disorders affect approximately 75% of persons with MS, with altered coordination of posture and gait [3, 4], mobility problems [5, 6], reduced walking competency [3] and increased risk of falling [7]. In progressive MS, the high prevalence of motor disorders and gait disabilities, the negative impact on personal activities and quality of life (QoL), and the limited effects of specific medications [8] make gait rehabilitation a crucial part of the management. The aim is to increase patients’ levels of activity and independence [9] and their QoL, even independent of symptom regression [10, 11]. Gait disabilities showed improvement following physical therapy [4, 12–14] and low-to-moderate-intensity aerobic over-ground or treadmill training, which represents a useful option for rehabilitation, also in combination with body weight support [15–17]. To this end, a robot-driven gait orthosis was recently developed, studied and considered a feasible and effective therapeutic option in MS subjects with severe walking disabilities. Robot-assisted gait training (RAGT) allows a more effective support of walking movements and imitation of a nearly normal gait pattern during treadmill training at a higher speed, with improvements in walking distance, velocity and knee extensor strength compared to conventional therapy [18, 19]. Several studies have tested in samples of MS patients the effects of interventions, such as treadmill training [4], bodyweight-supported training on a treadmill [20, 21], RAGT [18, 19, 22–26], or both treatments combined within a single session [27], reporting small but positive effects on functional status [4, 18–20, 23–26] or QoL [10, 21]. Recently, improvements were reported in the 6-min Walking Test and in the balance domain after RAGT [25] but not in gait speed measured by the 10-m Walk Test [26]. Unfortunately these studies, using different devices and training protocols (12 to 15 sessions over 3–6 weeks), including heterogeneous MS subgroups with a limited number of subjects and a wide range of gait disabilities (Expanded Disability Status Scale, EDSS 3–7.5), failed to offer an exhaustive evidence of the superiority of RAGT over other specific gait trainings, so larger trials are necessary [28]. Otherwise, this might be partially explained by the fact that the rehabilitation process in MS subjects is complex and person-specific [28] as the response to treatments regarding neuronal plasticity is highly individual. Functional recovery in MS is achieved by repair of damage through remyelination, with resolution of inflammation and functional reorganization. Evidence from brain systems supports an adaptive role for neuroplastic changes in MS despite its widespread pathology. Specifically, it may limit the negative effects of MS on behavior [29–32] and differs between patients and various disease types, with lower response according to patient age and disease duration [33, 34]. Moreover, different rehabilitation treatments might switch on different adaptive response. High-intensity interventions might be more effective on neural reorganization and motor recovery involving synaptic transmission and formation of novel synapses, cortical reorganization and induction of neurogenesis limited to the site of injury or involving distant healthy brain regions [35]. The effects of neuroplasticity-based technologies and interventions, virtually beneficial for functional recovery, have been poorly tested so far. Different tools, such as positron emission tomography and functional magnetic resonance imaging, could be appropriate to evaluate such recovery-related processes. Several studies have employed these techniques, revealing that in MS patients a decreased hemispheric lateralization [32] and an increasingly bilateral activation, even for simple motor tasks involving higher-control sensorimotor areas, were observed [36, 37]. Other noninvasive, reliable and less expensive measurements, such as transcranial magnetic stimulation and near-infrared spectroscopy (NIRS), could also be useful. In a NIRS-based study the coherence, considered a potentially useful marker in disorders with white matter damage or axonal loss, was found to be similar in MS subjects and controls in the resting phase, but significantly decreased during motor tasks [38]. Moreover, relevant information to identify patterns of recovery in MS patients could be added by the measurement of molecular regulators of neuronal or vascular plasticity. These biomarkers derived from blood tests include circulating cell subsets and soluble factors measurable in plasma. Previous studies involving MS subjects showed that the *N*-acetylaspartate concentration correlated with an increased lateralization index; neurotrophins that regulate neural plasticity, such as brain-derived neurotrophic factor (BDNF) [39], were found in lower concentrations compared to healthy subjects [40]. Furthermore, inflammatory cytokines, such as interleukin-1β, negatively interact with BDNF and amyloid-β has been observed in multifocal MS lesions [41, 42]. Finally, growth factors participate in neural cell survival and tissue repair processes [43–45] and, in particular, platelet-derived growth factor decreases with disease duration, being low in primary progressive MS patients [46] while incomplete glucose oxidation by glycolysis and mitochondria results in increased oxidative stress that promotes lesion progression rather to repair [47–49]. Also, disturbances of the hemostatic mechanisms, which are closely and reciprocally related to inflammation, are relevant for neurological disorders, in particular procoagulant factors and receptors, as well as main anticoagulant proteins endowed with anti-inflammatory activities, that exert cytoprotective effects and favor endothelial barrier stabilization, neurogenesis and angiogenesis [50]. Lastly, besides soluble factors, growing evidence advances the concept that stem cells can modulate nervous system action as well as the dysregulation of inflammatory responses and immune self-tolerance has to be considered a key element in the autoreactive immune response in MS. To this end, circulating stem/progenitor cells capable of homing in on neovascularization sites [51], and regulatory T-cells (Treg) have emerged as crucial players in the pathogenic scenario of autoimmune inflammation with a role in their modulation by pharmacological and rehabilitation therapy.

### Aims

The primary objective of the study is the verification of the starting hypothesis that RAGT could have greater benefit, compared with conventional therapy alone, in gait speed improvement as assessed by the Timed 25-Foot Walk Test (T25FW).

Secondarily, the study aims to determine whether fatigue, QoL, balance and mobility are improved by RAGT. Finally, the study aims to collect information on whether RAGT or the active control group (CT) influence markers of plasticity, including clinical and circulating biomarkers, and if these modifications are correlated with clinical outcome. The ultimate goal is to draw tailored rehabilitation strategies capable of bypassing the person-specific treatment response in MS patients.

## Methods

### Study design and setting

**Fig. 1:**
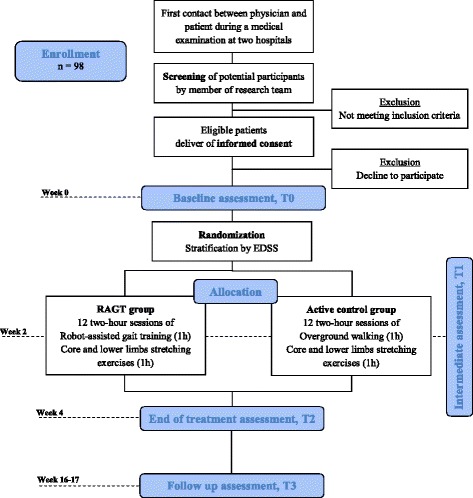
Design of the RAGTIME Study. *EDSS* expanded disability status scale, *RAGT* robot-assisted gait training

This study is a parallel-assignment, single-blinded, randomized controlled trial with the assessor blinded to the group allocation of participants (Fig. 1). Participants meeting the inclusion criteria and who provide written informed consent will be randomly assigned to one of the two treatments: the RAGT or the CT group. This study was approved by the Ethics Committee of Ferrara province with approval number 101-2012. This protocol is reported following the Standard Protocol Items: Recommendations for Interventional Trials (SPIRIT) guidelines [52]. A populated SPIRIT Checklist is available as additional file (Additional file 1). Subjects will be recruited using several methods, including the identification of MS patients who are referred to the Rehabilitation Clinics of the Operative Unit of Physical and Rehabilitation Medicine of Ferrara University Hospital and from the Centro Malattie Rare e Neuroimmunitarie, IRCCS Neuroscienze Bellaria, Bologna. All the interventions scheduled, as well as the outcome measure assessment, will be performed at the Operative Unit of Physical and Rehabilitation Medicine of Ferrara University Hospital.

### Selection criteria and recruitment of participants

Patients affected by primary and secondary progressive MS [53] will be invited to participate if they meet the following inclusion criteria:

- Men and women, aged 18 to 65 years
- Severe gait impairments, defined by an EDSS score [54] ranging from 6 to 7
- Ability to perform the T25FW
- Lack of MS worsening in the 3 months just before the intervention period
- Cognitive functioning to give informed consent identified by a Mini Mental Status Examination (MMSE) score ≥24/30 [55]

Exclusion criteria include:

- Neurological conditions in addition to MS that may affect motor function and other medical conditions likely to interfere with the ability to complete the study protocol safely, independently from the group assignment
- Considerable muscle spasticity, defined by a Modified Ashworth Scale (MAS) [56] score >3 or contractures that may limit range of motion or function of hip, knee or ankle flexors/extensors
- Relapsing of MS-related conditions or changes in drug therapy (both disease-modifying or symptomatic therapies) or any other confounding factor during the study
- Rehabilitation treatments or botulinum toxin injections during the 3 months preceding the start of the study

During the first meeting with potential participants the physician will ask them if they are interested in taking part in the study; if they are, the physician will address them to a specific screening visit to verify compliance with the inclusion criteria. If compliance is satisfactory, the study physician responsible for inclusion will deliver the letter explaining the study, as well the Consent Form, to the potential participant, and will encourage them to ask any question. After at least 3 days, the patients will be contacted by phone and asked about their decision; in case of voluntary participation, patients will be given an appointment where they will consign the informed consent and where a physiotherapist will perform the baseline outcome measures; if the patients have not yet decided, they will be given adequate time to consider their participation; whereas if the subjects decline participation, they will be thanked for their consideration. According to the Consolidate Standards of Reporting Trials (CONSORT) guidelines [57], the total number of screened subjects who are ineligible (and the reasons for their ineligibility), or who are not willing to participate in the study, will be tracked. To optimize recruitment, all members of the rehabilitation unit of the hospital, including physicians, physiotherapists and nurses, will be contacted weekly by the research coordinator to identify possible participants. Moreover, the research coordinator will participate in the rehabilitation team meetings to check the availability of potentially eligible patients. Information on the study procedures will also be given to the MS support groups.

### Randomization and blinding

After the collection of the informed consent and of the baseline data, the physician responsible for the enrollment will create the allocation sequence, on a personal password-protected computer. The password to log into the allocation list will be given only to the research coordinator, to the physician who created it, and to the administrator responsible of randomization. Then patients will be randomized to one of the two groups by the external administrator not involved in the trial to prevent selection bias through a computerized randomization stratification approach. Participants will be stratified by their degree of impairment (EDSS score) to obtain a balance between groups regarding the baseline physical capacity. The randomization scheme (1:1 ratio) will be set up in permuted blocks of 4 to ensure a similar number of participants between groups. Finally, the subjects will be assigned to one of the two treatment groups: RAGT (experimental group) or CT (active control group). The participants cannot be blinded to the two interventions as the two training protocols were detailed in the informative form for the patients given during the screening visit. Once randomized, patients cannot change the treatment assigned for any reason (e.g., participant request), but in case any medical conditions derived from that specific treatment develop, the training will be immediately suspended. Outcome measure assessors who have to remain blind to group allocation will not have access to the randomization list. Unblinding will not be permissible for these researchers. All outcome data will be recorded on two different electronic spreadsheets by two blinded researchers involved in the trial, and the accuracy of the data will be checked by the research coordinator for all the outcome measures. The privacy of the participants and their personal medical records will be guaranteed by treating the data according to the Italian Law n. 196/2003, to the “Safe Harbor Act” (2000/520/CE) and to the “European Union Data Protection Directive (95/46/EC 24 October 1995).”

### Intervention

Participants in both intervention groups will receive twelve 2-h training sessions over a 4-week period, resulting in a three sessions/week pattern. A pragmatic window will be set to ensure for each participant the execution of all 12 sessions in a maximum of 5 weeks, to accommodate possible withdrawal of one or more sessions (e.g., intercurrent illness, sudden family problems, etc.). Patients who miss more than three consecutive training sessions will be dropped out from the study. If a participant misses a training session, the physiotherapist will inform the research coordinator and will contact the patient by phone, to determine reasons for missing and to motivate them to take part to the next scheduled training session.

The first training hour will consist of the specific training scheduled for each group (gait training), whereas the second training hour will be common to both groups. In detail, an experienced physiotherapist will perform lower-limb and core stretching exercises to increase muscle flexibility as well as strengthening exercise for the lower limbs. The second training hour will be carried out over the entire duration of the study by the same physiotherapist, treating two participants contemporaneously, to ensure the same exercises for all patients to avoid possible confounding factors and bias during the results interpretation.

### RAGT experimental group

Patients included in this group will perform RAGT on the Lokomat treadmill (Hocoma AG, Volketswil, Switzerland). During these sessions subjects will wear a harness attached to a system to provide body weight support and they will walk on the treadmill with the help of a robotic-driven gait orthosis. The legs are guided according to a physiological gait pattern. The torque of the knee and hip drives can be adjusted from 100 to 0% for one or both legs. The speed of the treadmill can be adjusted from 0 km/h to approximately 3 km/h and body weight support from 0 to 100%. During the first session, these training parameters will be set according to subject characteristics and demand level. As training progresses, adjustments in the assistance provided by the driven gait orthosis (guidance), the amount of body weight support and treadmill speed will be performed. Training sessions will last for an hour with 30-min of real walking time because subject setup in the device takes approximately 30 min. At the end of each session the total meters walked, the average walking speed and the percentage of body weight support from 0 to 100% will be tracked.

### Active control group (CT)

Patients included in the CT will focus their efforts on gait training. During the 1-h individual physiotherapy sessions, patients will perform assisted over-ground walking for a total of around 40 min, inserted between a 10-min warm-up and cool-down period. The patient will be encouraged by the same experienced physiotherapist to walk back and forth on an 80-m indoor flat corridor with their habitual walking device (crutches, rollator). Every patient will be asked to walk without stop until reaching an effort corresponding to a value of 8 out of 10 of the Borg Rating of Perceived Exertion Scale [58]. When that given intensity has been reached, the patients will be allowed to rest sitting on a chair; after a suitable rest period, sufficient for the patient to return to values of 1/10 of the scale, the training will restart following the procedures previously reported. At the end of each session, the total meters walked, as well as the effective walking time, will be recorded on a properly developed module.

### Concomitant care and recommendations

During the 4-week period of in-hospital training, patients will be asked not to undertake other physiotherapist treatments. Moreover, patients will be asked to wear the same shoes and orthosis during all the testing and training sessions.

### Intervention fidelity and monitoring of adverse events

Before the beginning of the study, treatment physiotherapists will be trained by a member of the research team with a high level of experience in RAGT and CT training of MS patients. Physiotherapists will be provided with 1:1 guidance focused on the treatment’s characteristics and peculiarities, and their ability to complete training sessions will be verified. When the study begins, to each physiotherapist will be given the module to record the specific training administered to each participant. During the whole duration of the study, the members of the research team and research coordinator will randomly visit training sessions, to ensure that the scheduled intervention is being performed accurately and with high adherence to the protocol proposed. Any adverse unpredictable event will be recorded in the registry of each patient and the electronic database of the study, and managed according to the policies of the hospital, with referral for appropriate medical follow-up.

### Outcome assessment and data collection

Outcome measurements will be evaluated at the Operative Unit of Physical and Rehabilitation Medicine of Ferrara University Hospital by the same blinded assessors at the four time points (Fig. 1): (a) baseline (prior to the first intervention session, T0), (b) intermediate (after six training sessions, T1), (c) end of treatment (after the completion of 12 sessions, T2) and (d) follow-up (after 3 months from the end of training program, T3). Before the last outcome assessment, a blinded physiotherapist will contact each participant to remind them to attend the measurements sessions. A summary of the measures to be collected is reported in Table 1. A physician member of the research team involved in the subject enrollment will administer the MMSE and all the other clinical measures (EDSS, MAS) to determine eligibility for each patient. Moreover, the person responsible for the participant selection will record the general demographic information (age, gender), comorbidities and medical history.

**Table 1:**
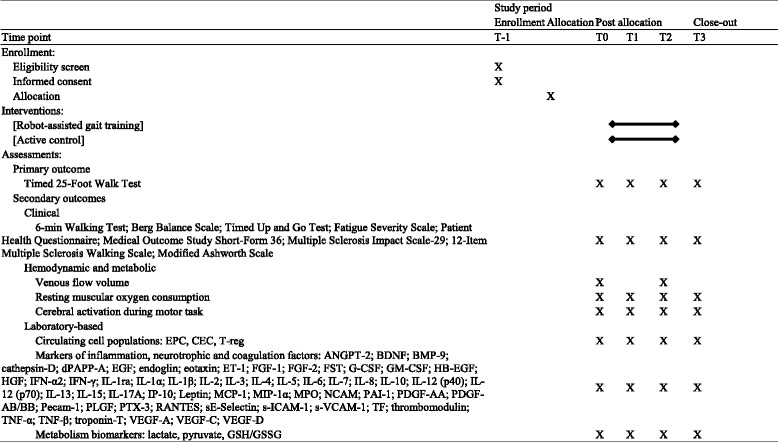
Schedule of enrollment, interventions and assessments

*Abbreviations*: *T-1* enrollment, *T0* baseline, *T1* intermediate, *T2* end of treatment, *T3* 3-month follow-up, *EPC* endothelial progenitor cell, *CEC* circulating endothelial cells, *T-reg* immunological rare cell populations, *ANGPT* angiopoietin, *BDNF* Brain Derived Neurotrophic Factor, *BMP* Bone Morphogenetic Protein, *dPAPP-A* Pregnancy-associated Plasma Protein A, *EGF* Epidermal Growth Factor, *ET* endothelin, *FGF* Fibroblast Growth Factor, *FST* follistatin, *G-* granulocyte, *GM-* granulocyte-macrophage, *CSF* Colony Stimulating Factor, *HB-EGF* Heparin-binding EGF-like growth factor, *HGF* Hepatocyte Growth Factor, *IFN* interferon, *IL* interleukin, *ra* receptor antagonist, *IP-10* 10 kDa-interferon gamma-induced protein, *MCP-1* Monocyte Chemoattractant Protein 1, *MIP-1α* C-C motif chemokine 3, *MPO* myeloperoxidase, *NCAM* Neural Cell Adhesion Molecule, *PAI* Plasminogen Activator Inhibitor, *PDGF* Platelet-derived Growth Factor, *Pecam* Platelet and Endothelial Cell Adhesion Molecule, *PLGF* Placenta growth factor, *PTX* pentraxin, *RANTES* C-C motif chemokine ligand 5, *sE-Selectin* Soluble Endothelial Leukocyte Adhesion Molecule, *S-ICAM* Soluble Intercellular Adhesion Molecule, *s-VCAM* Soluble Vascular Cell Adhesion Molecule, *TF* Tissue Factor, *TNF* Tumor Necrosis Factor, *VEGF* Vascular Endothelial Growth Factor, *GSH/GSSG* reduced glutathione/oxidized glutathione ratio

### Primary outcome: gait speed

This will be assessed by the T25FW [59] a quantitative measure of gait speed, also included in the Multiple Sclerosis Functional Composite. The patient is directed to one end of a clearly marked 25-ft course and is instructed to walk 25 ft (7.62 m) as quickly as possible, but safely, using the prescribed assistive devices. The task is immediately administered again by having the patient walk back the same distance. The test will be performed according to the instructions reported on the manual of the National Multiple Sclerosis Society. To ensure an accurate measure of the primary outcome, the time needed for each subject to complete the test will be collected with the aid of a photocell system (Cronopics, Chronojump Boscosystem Technologies, Rieti, Italy) with a precision of a millisecond and simultaneously by the assessors with a chronometer. Moreover, this test will always be performed as the first outcome measure, to avoid possible muscle fatigue derived from the other test previously taken. The walking speed will be calculated as the mean of the two trials performed.

### Secondary outcome measures

A significant number of secondary outcome measures will be employed to explore fatigue, QoL, balance, walking endurance, mobility and their possible connection with brain plasticity. Secondary outcomes will include: (1) clinical measures and questionnaires, (2) hemodynamic and metabolic evaluations and (3) laboratory-based measures. Every specific measure is detailed in the following section.

### Clinical and quality of life measures

1. 6-minute Walking Test (6MWT): walking endurance is measured with the 6MWT. This test, first validated in subjects with cardiopulmonary problems, is also considered a feasible, reproducible and reliable measure in MS [60]. Subjects will be instructed to walk up and down as far as possible on a 22-m walkway in 6 min without encouragement, with the possibility to slow down and rest if necessary. The total distance walked will be recorded
2. Berg Balance Scale: this is an assessment scale of ability to maintain balance – statically or while performing functional movement. It includes 14 observable tasks common to an everyday life measured on a 5-point ordinal scale [61]. This scale was previously widely employed in MS subjects [62]
3. Timed Up and Go test: the test is a combined measure of gait speed, balance, coordination and lower-limb strength. Subjects will be given verbal instruction to stand up from a chair, walk 3 m at their regular pace, cross a line marked on the floor, turn around, walk back and sit down [63]. The patients may use any gait aid that they normally use during ambulation, but may not be assisted by another person; however, a study staff member will guard the subject during the test. Subjects will perform three trials and the time it takes to perform each trial will be recorded with a stopwatch. The best trial will be considered
4. Fatigue Severity Scale: this is a method of evaluating fatigue in MS and other conditions. Essentially, the scale consists of answering a short questionnaire that requires the subject to read each statement and rate his or her level of fatigue from 1 to 7, depending on how appropriate they felt the statement applied to them over the preceding week [64]
5. Modified Ashworth Scale: this is a 6-point measure of spasticity; the assessor will rate the perceived amount of resistance or tone at the hip, knee and ankle flexor and extensor muscles [56]
6. Patient Health Questionnaire: this is a questionnaire to investigate the presence of depressive symptoms as well as to characterize the severity of depression [65]. It is composed of nine items based on the frequency of occurrence in the past 2 weeks of depression symptoms and has been found to be valid also in individuals with MS [66]
7. Short Form Health Survey: this is a generic measurement to measure health‐related QoL. It consists of eight subscales with a score ranging from 0 to 100 used separately as outcome measures of various aspects of health‐related QoL. It also measures two main health concepts: physical and mental. The questionnaire has been already employed in clinical studies involving MS subjects [67]
8. Multiple Sclerosis Impact Scale: this is a 29‐item self‐report measure with 20 items associated with a physical scale and nine items with a psychological scale [68]. Items ask about the impact of MS on everyday life in the past 2 weeks with possible answers set on a 1-to-5 Likert scale. Each of the two scales is converted to a 0–100 scale where a score of 100 indicates a greater impact of disease on daily function
9. Multiple Sclerosis Walking Scale: this is a 12-item walking scale that assesses a self-report measure of the impact of MS on the individual’s walking ability [69]

### Hemodynamic and metabolic evaluations

Complete brain circulation assessment will be performed by the application of a validated model with parameters measured at the bed side by the means of an Echo Color Doppler. Subsequent post analysis permits us to obtain in-flow and out-flow measurements as well as measurements of the rate of collateral cerebral venous return [70]. Metabolic measurements will be assisted by the NIRS technology and by assessment of circulating metabolic marker (refers to Laboratory-based measures section). NIRS is a noninvasive, portable technique for the ambulatory, remote monitoring of oxygenation changes in response to motor tasks of human muscles and brain cortex. Patients enrolled in the study will undergo metabolic evaluation consisting of different measurements: assessment of muscular oxygen consumption at gastrocnemius at rest (rmVO_2_) and dynamic evaluation of cerebral activation during a simple walking task performed on a treadmill.

#### Resting muscular oxygen consumption at gastrocnemius

The muscle metabolism assessment will be performed by a continuous wave system (Oxymon MK III Artinis Medical System, the Netherlands) consisting of two channels. The measurement, already employed in MS subjects [71], is performed on gastrocnemius in a resting supine position as detailed elsewhere [72]. The absolute value of rmVO_2_ will be measured in both legs and calculated by analyzing the rate of increase in deoxygenated hemoglobin concentration during venous occlusion. The concentration changes of deoxygenated hemoglobin will be converted into milliliters of oxygen per 100 g of tissue per min. The mean value between the two legs, as well as the data for the more impaired leg, will be considered for data analysis.

#### Cerebral activation analysis during a motor task

The brain metabolism evaluation will be performed using another optical imaging system (NIRScout, NIRx Medical Technologies LLC, Glen Head, NY, USA) consisting of 16 light source fibers and 16 detector fibers, resulting in a 48-channel recording of cortical changes in oxygenated, deoxygenated and total hemoglobin. For the measurements of the present study the optodes will be tightly placed on the skull with the use of a holder cap with the interoptode distance set at 30 mm, covering the bilateral motor and premotor cortex. Patients will walk on the treadmill at a speed of 0.2 km/h assisted by personnel and with partial body weight support if needed, performing four short tasks (30 s of walking) alternated by rest periods (30 s) [73]. Data will be analyzed for possible selective changes of cerebral perfusion by specific software (NIRSlab). For each patient will be calculated the area under the curve of oxygenated, deoxygenated and total hemoglobin for each channel and hemisphere (media of the area under curve of each one of the 24 channels of selected hemisphere). Moreover, the data will also be analyzed with the software NIRS-SPM to draw a map of activation of the selected brain areas during the test and to perform a *t* test statistical comparison within subjects and between treatments, as well as other possible correlations with clinical or laboratory parameters.

### Laboratory-based measures

#### Samples processing (harvesting and storing)

Circulating biomarker analyses will be performed on blood samples collected from patients at each time point (T0, T1, T2 and T3), in fasting conditions, at the University Hospital of Ferrara. Blood collection will consist of a total of approximately 18 ml of whole blood distributed in three different test tubes (based on the analyses to be performed) containing either ethylenediaminetetraacetic acid (EDTA) or sodium citrate or ribonucleic acid (RNA) stabilizer. Once a suitable vein has been identified, the area where the needle will be inserted will be sterilized and the needle inserted. When the amount of blood extracted satisfies the study requests, the needle will be removed from the vein and a swab will be placed on the forearm. The patient must remain in a resting position for few minutes and they will be asked to communicate any adverse reaction felt. The blood samples collected will be labeled with a unique alphanumeric code identifying each participant; samples will be then packed and safely transferred to the Biobank Service of the Laboratories of the Technologies of the Advanced Therapies of the University of Ferrara. Samples collected in EDTA will be processed in real time for rare circulating cell population quantification through multiparametric flow-cytometry analyses. Samples collected in sodium citrate will be centrifuged and plasma will be collected, aliquoted and frozen at −80 °C in multiple and single-use aliquots for analysis of soluble circulating factors. Samples collected in RNA stabilizer will be used for analysis of transcriptional expression profiles. The biological samples of the participants will be catalogued and stored anonymously using the unique alphanumeric code identifying the patients, the access to samples will be restricted to the researchers involved in the RAGTIME trial.

#### Blood samples analyses

- Circulating progenitor cell subsets, circulating endothelial cells and immunological rare cell populations (Treg) will be evaluated in fresh blood collected in EDTA by multiparametric flow-cytometry analyses [74, 75]
- Levels of glycolytic or mitochondrial activities will be obtained by analysis of the metabolic markers lactate and pyruvate and by the Glutathione Ratio. These will be determined in plasma samples by colorimetric assays [76–78]
- Cytokines/chemokines/growth factors, neurotrophic factors, markers of brain damage and coagulation factors will be quantified in plasma samples by multiplex immunoassay according to the manufacturer’s instructions by using specific Milliplex MAP kits (Luminex xMAP technology, Merck-Millipore, Germany) or by commercially available enzyme-linked immunochemical and immunosorbent assay following the manufacturing instructions [76, 79, 80]
- Additional plasma proteins, encoded by RNA selected by transcriptomic approaches [81], will be measured in plasma samples by the above reported assays
- Metabolism markers (lactate, pyruvate and Glutathione Ratio) will be determined in plasma samples by colorimetric assays [82]

Additional information is reported in Table 1.

### Data management

The statistical unit will be responsible for data management (quality control and data cleaning) and data analyses according to the different research hypotheses described. A statistician will be in charge for the analysis of clinical and laboratory data, using several statistical packages such as Medcalc Software (MedCalc Software bvba, Ostend, Belgium), IBM-SPSS Statistics (IBM, Armonk, NY, USA) and Stata Statistical Software (Release 13. College Station, TX: StataCorp LP, USA). Study design and statistical plan of the study has been discussed with, and prepared by, the supporting statistical unit working at our institution.

### Sample size and power

The primary outcome for this study is to detect walking differences (specifically, T25FW measurement) between MS subjects who underwent RAGT and MS subjects who underwent CT. To calculate the sample size for this study, we used the data from a previous study [18] where a RAGT effect size of 0.40 was observed. This value is based on a decrease of values for T25FW from 8.8 ± 3.1 s at baseline to 7.4 ± 3.8 s after RAGT. Given equal allocation between treatment and control arms, 88 subjects are required to maintain a Type I error rate of 5.0 and 80% power to detect a difference between intervention groups. Conservatively, we expect a 10% rate of dropout or loss to follow-up. We will assume that dropout participants will not improve from the last measured point; thus, the sample size will be increased by 10% to 98 subjects (49 per arm).

### Statistical analyses

Standard methods for the analysis of randomized controlled trials will be employed. Firstly a comparison of baseline characteristics of the two groups will be compared for demographics, primary and secondary outcome measures. Though our starting hypothesis called from a superiority of RAGT with respect to CT, to allow for the chance that participants enrolled in the CT group could have better outcomes than those in the intervention arm, we will use two-tailed tests of significance for all analyses. A *p* value of 0.05 will be considered statistically significant.

For the primary hypothesis, a two-way repeated measure analysis of variance (factors: treatment, time) will be run to compare differences in gait speed within the RAGT and CT groups at baseline, after treatment and follow-up. Given the large number of planned secondary outcomes, in order to reduce the probability of a false positive result, for these specific analyses the significance level will be fixed at 0.01.

### Additional statistical analysis

The statistical significance of primary and secondary outcome measures in score change between the groups will be assessed using *t* tests for symmetrically distributed data and analogous nonparametric tests, such as the Wilcoxon signed-rank test, for data that are skewed. If a statistically different distribution between the two groups in the baseline outcome level is identified, a secondary analysis that employs a multivariate modeling, such as analysis of covariance, to adjust for these factors, will be performed. Since MS encompass a wide spectrum of disabilities, the general linear model will also analyze the effect of several factors, so we will conduct exploratory analyses in which we will include the MS onset (year) and EDSS subscores (pyramidal function, cerebellar function and sensitive functions) which define our MS in the model. Furthermore, the possible effects of disease-modifying or symptom-modifying drugs (i.e., fampridine) on motor outcome will be explored. Permutation tests may be implemented to verify that valid *p* values will be obtained even if model assumptions are not correct. Analysis of hemodynamic, metabolic and laboratory-based measures will be performed according to the procedures detailed above. Possible correlation between these factors and clinical measures will be assessed by Spearman’s rho or included in multiple regression models.

### Intention-to-treat

All analyses will be conducted using intention-to-treat, where any subject randomized to one arm remains in that arm regardless of whether or not they received the intervention. Missing values, though we will make any effort possible to reduce their incidence, will be treated using the multiple imputation procedure, considered one of the best methods to handle missing data [83]. Moreover, a sensitivity analysis that assesses the stability of the study’s conclusions, comparing intention-to-treat analysis to an analysis that takes into account level of participation in the intervention arm, will also be performed.

### Data monitoring and interim analysis

The RAGTIME trial, dealing with well-established and safe rehabilitation procedures, paralleled by a rigorous handling of potential harms as a local hospital policy, expects to minimize all the potential risks. Moreover, this being a single-center study, a Data Monitoring Committee will not be required. The research coordinator will be in charge of the interim analysis (with statistician support) and for taking the final decision to stop, modify or terminate the trial. Eventual possible modifications or amendments to the protocol will be discussed within the research group and communicated by the research coordinator to the funding body for approval or refusal. An interim analyses performed by the scientific experts of the funding body will be scheduled after 18 months from the beginning of the trial. It will consist of a compilation of an interim evaluation checklist form, of a study interim report form and a public preliminary data presentation and discussion. Once the trial is concluded, the research coordinator will be responsible for the final dataset, and will state the number of research team members who can have access to the data collected.

### Dissemination plan

The results derived from this trial will be published in high-quality journals and presented at national and international meetings. The results will also be disseminated through conferences organized by the funding agency, Regione Emilia Romagna. The authors also intend to spread the information learned from this project by presenting it at MS support groups, to inform the patients about the efficacy of one or the other treatment and the local availability of them.

## Discussion

The study may provide significant information on the rehabilitation of severely disabled, progressive MS patients, clarifying the possible application of technologies in clinical practice with a high number of participants with a limited range of disability; thus, reducing the bias present in the previous studies.

From the proposed trial, we expect to observe a greater effect of high-intensive robotic rehabilitation on mobility and functional recovery in a large cohort of MS patients compared to CT. Functional recovery after rehabilitation programs has to be considered a multifactorial process in which integrated and multicomponent biological systems are implicated, i.e., vascular, neuronal and metabolic. Quantity (duration and frequency) and quality (task-specificity) of interventions are appropriate to facilitate enhanced neural reorganization and motor recovery, favoring adaptations in the adult brain. Cortical adaptive changes could, therefore, contribute to functional recovery from lesions and may have an important role in compensating for axonal injury in MS. Therefore, following the intervention or during the different phases of intensive rehabilitation, we expect to detect possible modulation of biomarkers of brain plasticity related to cortical activation and/or of circulating regenerative markers, potentially correlated with the clinical outcomes. Our findings will help to identify specific markers to detect whether a patient should be a “responder” to a rehabilitative intervention, bearing in mind that due to a wide variety of symptoms, the rehabilitation process in persons with MS is multifactorial and should always be tailored to patient characteristics. Moreover, this study might increase knowledge on the effects of MS gait rehabilitation, leading to an optimization of health care resources and developing cost-effective rehabilitation programs. Lastly, from a scientific point of view, the study represents a step towards the knowledge of the functional reorganization processes in progressive MS patients, identifying the effectiveness of intensive rehabilitative interventions through the changes of clinical and circulating biomarkers of MS plasticity. However, further studies will be necessary to confirm the results related to the secondary outcomes.

### Trial status

Recruiting.

## Additional file

Additional file 1: SPIRIT filled checklist. (DOC 124 kb)

## Abbreviations

6MWT: 6-min Walking Test
BDNF: Brain-derived neurotrophic factor
CT: Conventional therapy
EDSS: Expanded disability status scale
EDTA: Ethylenediaminetetraacetic acid
MAS: Modified Ashworth Scale
MMSE: Mini mental status examination
MS: Multiple sclerosis
NIRS: Near-infrared spectroscopy
QoL: Quality of life
RAGT: Robot-assisted gait training
rmVO_2_: Resting muscular oxygen consumption at gastrocnemius
T25FW: Timed 25-foot Walk Test
Treg: Regulatory T-cells
